# The Billings Ovulation Method and Catholic Women’s Health Activism in 1970s and 1980s Ireland

**DOI:** 10.1093/shm/hkag078

**Published:** 2026-09-17

**Authors:** Laura Kelly

## Abstract

This article explores the history of the Billings Method, a form of ‘natural’ family planning, in Ireland from its initial popularisation in the 1970s to the beginning of its decline in the late 1980s. Drawing on oral history interviews with women who taught the method in Ireland in this period, as well as previously unexplored archival sources, the article seeks to nuance understandings of conservative women’s activism in this period. It argues that the Billings movement had much in common with the tenets of feminist health activism. However, promoters of the method ultimately failed to garner support from members of the Irish medical profession and Catholic Church hierarchy.

The Billings Ovulation Method (later Billings Method for brevity), is a method of ‘natural’ family planning which was pioneered by the Australian married couple, Dr. John Billings and Dr. Evelyn (Lyn) Billings in the 1950s and 1960s.^1^ The method relies on the observation and charting of changes in cervical mucus to detect when ovulation occurs and avoidance of sexual intercourse on potentially fertile days. During ovulation, the cervical mucus becomes clear, slippery and elastic, like the consistency of egg whites. Through careful use, the method can be used to avoid conception or to plan a pregnancy. The Billings Method was first introduced to Ireland in the early 1970s when Father Dermot Hurley, an Irish Columban priest in Fiji and promoter of the method was home on holidays in Cork visiting his sister, Noreen Ryan and discussed the method with her.^2^ Hurley had first learnt of the method during a visit to Drs. John and Lyn Billings in Melbourne, Australia in January 1971.^3^ On his return to Fiji, he had it taught in his local area.^4^ Following correspondence with the Billings in Australia, Noreen Ryan gave a talk to a women’s club in Mayfield, Cork, and after that began to hold meetings twice a month there for couples who were interested in the method. By 1973, Ryan and other volunteers were running six different centres in Cork and had personally financed the printing of a taped lecture by John Billings on the method.^5^

Concurrently, Dublin woman Mavis Keniry, the mother of two small children, had also discovered the method in the early 1970s. In an interview with the *Sunday Independent* in 1978, Keniry explained that she had become interested in the method because of her own personal experiences. Keniry was married at 21 and after five years, became pregnant but lost her baby at seven months' gestation. Two years later, she gave birth to her daughter and began ‘her long search for some safe, acceptable means of contraception’. Through her research, Keniry was aware of the side-effects associated with the contraceptive pill. When she first heard about the Billings Method, she sent away for relevant literature and finding that the method worked for her, ‘she spread the word to many of her friends, most of whom were then young mothers in their twenties’. Following this, Keniry and her group of friends established the Dublin Ovulation Method Advisory Service (DOMAS).^6^

This article explores the history of the Billings Method in Ireland through the experiences of the women who promoted it in the 1970s, 1980s, and 1990s. The health activism of these teachers should be placed within the wider context of Catholic theology and practice in the second half of the twentieth century, particularly in relation to ideas around the ‘lay apostolate’, Catholic understandings of sexuality after the Second Vatican Council (1962–5), and recent theoretical framings of ‘lived Catholicism’. Prior to the Second Vatican Council, the concept of ‘Catholic Action’, whereby the laity undertook initiatives to address social and moral concerns of the early twentieth century; this included formal organisations in the Irish context such as the Legion of Mary (1922).^7^ After the issue of the 1965 decree *Apostolicam Actuositatem* (Apostolic Activity) as one of the magisterial documents of the Second Vatican Council, under Pope Paul VI, the lay apostolate was given a ‘fresh theological impetus’.^8^ The work and activism of teachers of the Billings Method ought to be understood within this framing and as a response to this call.

The Catholic Church had been seriously concerned with the issue of birth control since the 1920s.^9^ In 1930, the papal encyclical *Casti Connubii* (of chaste wedlock) was published by Pope Pius XI.^10^ The encyclical emerged in response to the Lambeth Conference of 1930 where the Anglican bishops had passed a resolution that birth control might be acceptable in some instances.^11^  *Casti Connubii* denounced any form of birth control, condemned eugenics and abortion, and reasserted the main aim of marriage being to produce children.^12^ Almost forty years later, the 1968 papal encyclical, *Humanae Vitae*, which was issued by Pope Paul VI, reasserted this condemnation of all methods of ‘artificial’ birth control, representing in the words of historian Alana Harris ‘a “spiritual” crisis or “religious” reformation in a year of revolution’ in Europe.^13^ Prior to its publication, Catholics around the world had been expecting that the encyclical might represent a more relaxed attitude to the use of contraception. For individuals who wished to abide by Catholic Church teachings, only ‘natural’ forms of family planning could be used. This created a significant tension for Irish men and women who wished to use an effective form of family planning in line with Catholic teachings.^14^ Earlier forms of ‘natural’ methods such as the safe period, also known as the ‘rhythm method’, and the temperature method were notoriously unreliable so the Billings Method thus potentially offered a means for couples to more effectively plan the number of children they wished to have without resort to ‘artificial’ forms of contraception.^15^ The safe period or ‘rhythm’ method, which had been developed in the 1920s by Japanese gynaecologist Ogino Kyusaku and Austrian gynaecologist Hermann Knaus respectively and which was declared acceptable to the Catholic Church in 1951, was dependent on a regular menstrual cycle and could be extremely unreliable. The temperature method, developed by Catholic priest Wilhelm Hildebrand in the 1930s, linked the measurement of basal temperature to birth control, finding that at the time of ovulation, a woman’s temperature rose slightly meaning that with careful records, women could potentially detect ovulation and avoid sexual intercourse at this time. However, this method was also viewed by many women as restraining and undependable.

In relation to *Humanae Vitae*, scholars such as Alana Harris and David Geiringer have drawn attention to the nuances in its message. Geiringer has suggested that the encyclical ‘was not produced by a refusal to engage with the outside world or an ignorance of secular thinking. In some ways, the Commission’s case for change was undermined by its zealous adherence to contemporary secular understandings of sex’.^16^ In relation to ideas around ‘natural family planning’, the papal encyclical called on ‘men of science’ who ‘can considerably advance the welfare of marriage and the family, along with peace of conscience, if by pooling their efforts they labour to explain more thoroughly the various conditions favouring a proper regulation of births’. Pope Paul VI hoped that medical science would ‘succeed in providing a sufficiently secure basis for a regulation of birth, founded on the observance of natural rhythms’ and by doing so, ‘scientists and especially Catholic scientists’ would help to demonstrate the Church’s teaching that ‘a true contradiction cannot exist between the divine laws pertaining to the transmission of life and those pertaining to the fostering of authentic conjugal love’.^17^ In addition, *Humanae Vitae* did not just reiterate the Catholic Church hierarchy’s stance against ‘artificial’ contraception, but as Alana Harris has argued, it also developed ideas around ‘chaste limitation’, ‘companionate marriage’, masculinity and male sexual desire, spousal responsibilities, as well as touching on themes such as the importance of physical intimacy in marriage.^18^ Such ideas were expanded on by Pope John Paul II in his *Theology of the Body* (1979–84). There have also been recent calls encouraging scholars to utilise the theoretical framework of ‘lived Catholicism’, a development from ‘lived religion’, in trying to foreground the lived experiences of Catholicism. This involves attention to the differences in geographical experiences of Catholicism and, in the words of Tricia Bruce should ‘provoke the edges, trouble our sense of what is, and compel us to listen more closely to everyday manifestations of church in the world’.^19^

This article brings these different areas of scholarly enquiry into conversation with each other through an examination of the history of the Billings Method in Ireland from its initial popularisation in the 1970s to the beginning of its decline in the late 1980s. While there has been significant research into the history of contraception in Ireland and other geographical contexts in recent years, ‘natural’ methods such as the Billings Method have received limited attention.^20^ An important exception is work by the *Catholicising Reproduction, Reproducing Catholicism* group which has developed our understandings of Catholic approaches to family planning in Poland.^21^ More broadly, significant research in a range of geographical contexts has highlighted the tensions between Catholic beliefs and family planning practices after *Humanae Vitae*.^22^ In exploring the history of the Billings Method in Ireland and the experiences of the women who popularised it, this article seeks to build on interventions by scholars such as Caitriona Beaumont and Grace Heaton who have nuanced our understandings of the range of women’s activism in the late twentieth century and uncovered the histories of lesser-known activists.^23^ Relatedly, there have been a number of important studies of feminist health activism in relation to reproductive rights in Ireland in the 1970s and 1980s.^24^ However, with some valuable exceptions by scholars who have explored the history of the Irish anti-abortion movement, and in particular, the Pro-Life Amendment Campaign (PLAC), less attention has been paid to socially conservative activism, and in particular, the role of women in activist groups which were resistant to the social changes of this period.^25^ This article also seeks to contribute to wider understandings of the role of Catholicism in Irish women’s lives. Recent studies by Cara Delay and Lindsey Earner-Byrne have complicated understandings of Irish Catholic lay women in the first half of the twentieth century, showing how they were not passive actors.^26^ More broadly, the article seeks to contribute to recent international studies on the activism of conservative women.^27^ It also seeks to respond to Sylwia Kuzma-Markowska and Agata Ignaciuk’s call to scholars ‘to explore the Catholic family planning movement’s transnational relations and cooperation beyond *Humanae Vitae*’.^28^

I draw here on oral interviews with 13 teachers who taught the Billings Method in Ireland from the 1970s to 1990s, primarily in Dublin, Cork and Co. Louth, as well as the daughters of two key proponents of the method, in order to explore women’s personal experiences of teaching the method, their reasons for involvement, and their wider worldviews. This research was conducted as part of a wider Wellcome Trust-funded project entitled ‘Contraception and Modern Ireland, *c*.1922–92’. Teachers of the Billings Method were recruited for oral history interviews through contact with Billings Method Ireland, previously the National Association for the Ovulation Method Ireland, and snowballing.^29^ I also utilise a range of previously unexplored archival sources including the minute books of the Dublin Ovulation Method Advice Service (DOMAS), the papers of Mavis Keniry, and Irish newspapers from the 1970s and 1980s, accessed through digital databases.^30^ Through an examination of these sources, I discuss the history of the Billings Method in Ireland primarily from the perspectives of the lay teachers who promoted it.

I start by exploring the early history of the Billings Method in Ireland, situating it within the broader context of social change in the late twentieth century in the country, before going on to discuss the experiences and motivations of teachers of the method. The article then analyses the experiences of clients, how teachers defended the method, the tensions between teachers, the medical profession and Catholic hierarchy, and finally, links with the Irish anti-abortion movement. In addressing these themes, the article nuances understandings of conservative forms of activism in 1970s and 1980s Ireland. It argues that the Billings movement, through its rejection of medical forms of contraception, emphasis on woman-to-woman teaching, critiques of the male-dominated medical profession, and focus on empowering women with information about their bodies and reproductive cycles, could be seen as a bridge between feminist health activism and Catholic activism. Yet, for many women involved in the movement, the method, with its focus on a ‘natural’ method of family planning, and its foundations in early twentieth-century Catholic understandings of sexuality and marriage, represented a fulfilling form of lay apostolic work. However, promoters of the method ultimately failed to garner support from members of the Irish medical profession and Catholic Church hierarchy. A study of the Billings Method in Ireland is thus revealing of the tensions and constraints of Irish Catholic women’s activism in this period but also illuminates the transnational elements of this story.

## Ireland in the 1970s and the emergence of the Billings movement

The popularisation of the Billings Method must be understood within the backdrop of significant social and economic change in Ireland in the 1960s which built upon developments of the 1950s.^31^ Carole Holohan has argued that the sixties should be recognised as a period of ‘accelerated’ rather than ‘transformative’ change.^32^ Free post-primary education was introduced in 1967 and from 1966 to 1971, there was a fall in the number of young people in gainful employment and a rise in the number of those in education.^33^ For young people, the 1960s ‘marked a significant break with the immediate past’ due to falling rates of youth emigration, a rise in the number of young people engaged in agriculture and education.^34^ Sexual themes and issues such as contraception and divorce became an increasingly visible part of popular culture as a result of imported television programmes and popular music in 1960s Ireland.^35^ By 1963, there were 150,000 television licence holders in the Republic of Ireland. Imported programmes from Britain were more likely to normalise sexual themes and address issues such as contraception and unmarried motherhood.^36^

The issue of the legalisation of contraception, which had been banned in Ireland in 1935 under the Criminal Law Amendment Act, sparked wide debate in late 1960s and 1970s Ireland as a result of the agitation of family planning activists and feminist campaigners.^37^ The contraceptive pill had been available as a ‘cycle regulator’ in Ireland from 1963 while family planning clinics, which were established in major cities in the country in this period, got around the law by asking for a donation rather than charging a fee. For many men and Irish women, however, access to contraception was limited. In her study of Irish women’s relationships between their sexuality and the teachings of the Catholic Church, based on oral histories conducted with women in Cork in the mid-1970s and again in 2000, Betty Hilliard has ‘characterised this journey as one from domination, to resistance, to altered consciousness and, consequentially, changed practices. In this process the struggle of structure and agency can be discerned’.^38^

The 1970s was also an important period of debate in relation to women’s rights in Ireland. In this period, women suffered from a range of inequalities including lack of equal pay for equal work. The marriage bar, which meant that women employed in the civil service and as primary school teachers, had to leave their jobs upon marriage, was not lifted until 1973, unlike other geographical contexts where such marriage bars were lifted in the 1950s. Judith Harford and Jennifer Redmond have shown how, in relation to the marriage bar, ‘norms based on sexual differentiation in society were used to limit women’s careers and ambitions’.^39^ While the Irish marriage bar applied to women in the civil service and to primary school teachers, it was also widespread in local authorities, health boards, state-sponsored bodies such as Aer Lingus (Ireland’s airline), Córas Iompair Éireann (the national public transport provider), banks, financial institutions, and some private sector employers.^40^ The early 1970s also witnessed the advent of second-wave feminist groups such as the Irish Women’s Liberation Movement (1970) and Irishwomen United (1975) to campaign against these inequalities, as well as a number of conservative groups such as the Nazareth Family Movement (1970), Mná na hÉireann (1971) and the Irish Family League (1973), which were concerned with the changes in Irish society and focused their campaigns on moral issues such as contraception and abortion. In this period, the Irish Catholic hierarchy had begun to encourage individuals to follow their conscience in relation to moral issues, meaning that in the absence of Church leadership, such issues were taken up by conservative campaigners.^41^ These conservative groups later formed the foundations of the anti-abortion movement in Ireland. In relation to promoters of the Billings Method, Mary E. Daly has argued that ‘the lay leadership and the large numbers of women who were involved […] indicate a change in Catholic church involvement in socio-political campaigns, with the Hierarchy taking a less prominent role’.^42^

The 1980s and early 1990s represented a period of sexual repression in Ireland, particularly in terms of reproductive rights.^43^ However, the period was also witness to creative forms of activism in relation to reproductive and sexual health and LGBT rights.^44^ There were divisive referendums over divorce (1986, 1995) and abortion (1983, 1992).^45^ The 1983 public referendum which introduced the Eighth Amendment into the Irish constitution, effectively outlawing abortion came about because of the mobilisation of a number of anti-abortion groups such as the PLAC and the Society for the Protection of Unborn Children (SPUC).^46^ The following year, 1984, Ireland witnessed two distressing cases, which were seen to symbolise the repression of women in 1980s Ireland. The first was the death of Ann Lovett, a 15-year-old schoolgirl, who died after giving birth to a stillborn baby at a grotto in Granard, Co. Longford, in January 1984.^47^ The second was the Kerry Babies case, a 17-week public inquiry after two new-born babies were found dead within 100 km of each other in April 1984. Joanne Hayes, the mother who had concealed one of the babies, was arrested and charged with murder of the other baby, based on the erroneous claim that she was the mother of both.^48^ Young people were particularly affected by a lack of sex education and wider climate of stigma and shame in relation to sexual health.^49^

The emergence of the Billings groups in Dublin and Cork in the early 1970s therefore occurred at an important juncture in Irish social history, during which debates around sexual and reproductive health matters were divisive and during which it appeared that older, conservative ideals of ‘the family’ were under threat. The small group meetings in women’s homes in Dublin and Cork soon evolved into centres around the country where women could come and be taught the Billings Method. The work conducted by the teachers was entirely voluntary. At a May 1975 meeting of DOMAS teachers, it was agreed that a registration fee of 50p should be collected from each client who returned for a first reading.^50^ In 1978, the group charged a £1 donation for a book of instructions, chart and stamps.^51^ In 1979, it was proposed at a DOMAS meeting that ‘£2 be charged for Book, chart and stamps. Everyone agreed it was a good idea’, except for one teacher who feared it ‘it might be prohibitive for poor people’.^52^ All the consultations, however, were completely free. The teachers also actively tried to publicise the method. From 1975, notices were displayed on church notice boards in Dublin parishes.^53^ In 1979, they approached *Woman’s Way* magazine regarding an article on the Billings Method and produced new brochures, posters, and handouts, which were sent to members of the Church, government, medical profession, chemists, media, and health food stores.^54^ Teachers were also asked to ‘canvass their Drs. & priests re: O.M. and the availability of the service in their area’.^55^ Women often found out about the centres through word of mouth from friends or neighbours.

After an initial instruction at a centre, usually held on an evening in a parish hall or school hall, women commenced charting their cycle using a chart and stamps. The chart was ‘divided into spaces to denote the days of the cycle. The woman uses three lines of this chart for each cycle, one for the stamps and two to write descriptions (colour and consistency) of the mucus’. Three types of stamps were used, including red to denote the days of bleeding, green to denote the infertile days, and white, with a diagram of a baby, to denote the possibly fertile days’.^56^ Following this initial instruction, clients were met with at the end of the first cycle, and were seen again ‘at regular intervals until completely confident’.^57^ Figures 1 and 2 illustrate the fertile and infertile periods of the cycle.

**Figure 1.**
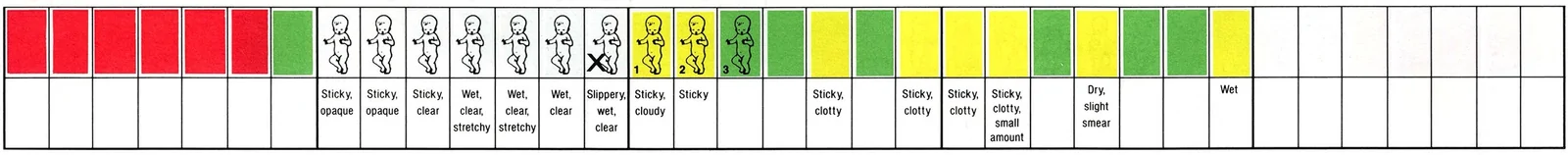
Diagram of a fertile cycle. Originally published as Figure 8 in Billings Atlas of the Ovulation Method, 5th Edition, 1989. First published in 1973. With permission from Ovulation Method Research and Reference Centre of Australia.

**Figure 2.**
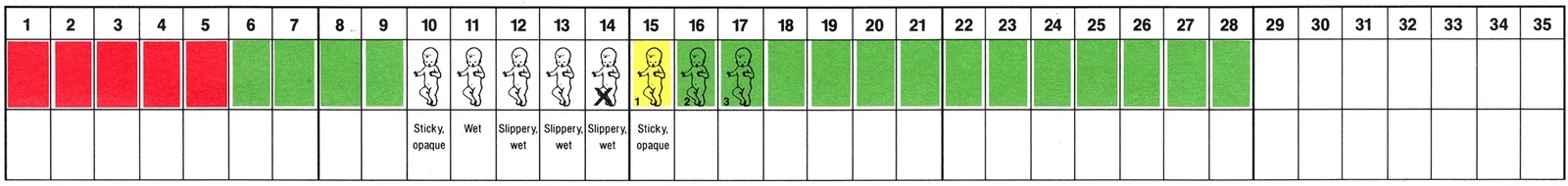
Diagram demonstrating the ‘peak rule’ and days of possible fertility. As denoted by diagram, baby stamps are used for days 10–17, a yellow stamp for the day of sticky, opaque mucus, and green stamps for the dry days. Originally published as Figure 18 in Billings Atlas of the Ovulation Method, 5th Edition, 1989. First published in 1973. With permission from Ovulation Method Research and Reference Centre of Australia.

In the 1970s and early 1980s, there were dozens of Billings centres around the country. By May 1975 there were Dublin centres in Inchicore, Ballyroan, Donaghmede, Balbriggan, Mount Merrion, with plans for centres in Rialto and Ballymun.^58^ By February 1978, the *Irish Independent* reported that there were 80 to 90 active groups in Ireland teaching the method.^59^ Ita recalled ‘When we started, we would have women sitting down on steps of stairs waiting to come in to have their chart read and they’d be a group in hearing it for the first time’.^60^ Statistics relating to numbers of clients are patchy, so it is difficult to determine exact numbers who attended the centres. In a newspaper article in 1980, Mavis Keniry stated that she believed over 20,000 Dublin women were using the method and that the number had doubled in the previous 3 years. She estimated that 50,000 women were using it throughout the country.^61^ There was certainly a demand for natural methods. A survey of 1158 members of the Irish Countrywomen’s Association in 1978, a year before the Family Planning Act, found that 80 per cent surveyed wanted some form of National Family Planning Service and of the women surveyed, 25 per cent said that ‘natural methods, and not artificial means of family planning ought to be promoted’.^62^ The minutes of the 1986 AGM reported that in 1985, 489 consultations had taken place at centres in the Northern Region, 996 in the Eastern Region, 632 in the North-Eastern region, 69 in the Midlands Region, 645 in the Southern region, a total of 2,831 consultations across the country.^63^

Early teachers had no formal training but once they became confident in the method, they soon set about organising 8-week courses. In October 1976, DOMAS began a teachers’ training course which took place over eight consecutive evenings at a cost of £5 per person.^64^ The course, according to Vera ‘covered all aspects of the method itself, it also covered communication skills, how to deal with one to one and small groups’.^65^ Once teachers completed their training, they were required to ‘feel confident with your own stamp chart’ before teaching others and should gain experience ‘by instructing no more than six couples or individual women. These six clients to be given follow-up help until more than confident’ and to work with a more experienced partner until they felt confident.^66^ In addition, teachers, or instructresses as they were called, ‘met once a month to share ideas and offer help to one another’.^67^ The DOMAS held their first meeting for teaching associates on 13 February 1975. Meetings generally took place monthly and enabled the teaching associates to discuss matters relating to the running of and opening of centres, numbers of new clients and chart readings, plans for promoting the method, and discussion of correspondence received. The teachers also reported on articles that mentioned the ovulation method in the press or interesting talks they had attended related to reproductive health. The group also occasionally had guest speakers who gave short talks at their meetings.

To be accredited, teachers were required to ‘accept the aims and objects of the Ovulation Method Research and Reference Centre of Australia (WOOMB Australia) and of WOOMB International’. These aims and objectives included the promotion of fertility control, using natural methods, to couples wishing to have children and couples wishing to avoid pregnancy, as well as ‘the rejection of contraception, abortion, and direct sterilization of the male or female’. Renewal of accreditation occurred ever 3 years.^68^ Anne recalled the training being ‘very well structured’ and that training occurred on a regular basis ‘because it was evolving and becoming more and more and more scientifically proven that it is reliable. And information is coming from Australia, particularly when John and Lyn Billings were alive’.^69^ Later, teachers were required to undertake an examination after which they would be awarded a certificate. Noreen recalled ‘It was quite strict. Like you were a teacher but you had to be validated all the time. You had to go to the teacher meetings and you had to go to the training sessions, some of which were in Cork, and make sure you were up to date with the very latest that was happening and, you know, anything that you could pass on to the clients’.^70^

Throughout the 1970s, DOMAS worked on becoming more professionalised. In May 1975, six members of the group attended a Communications Course run by the Catholic Church in Dublin.^71^ In June 1975, Keniry announced plans to establish a National Council for the Ovulation Method which ‘would cover all parts of Ireland, North & South, and would be a safeguard in preventing the method from falling into disrepute’.^72^ Keniry also drafted a Statement of Policy to be followed by teachers in July 1975 while in September 1977 a copying machine and rubber stamp were purchased for DOMAS ‘to simplify correspondence’.^73^ The constitution for the National Association of the Ovulation Method of Ireland (NAOMI) was passed at the group’s AGM in 1977. The aims and objects of the organisation included ‘to serve God by preserving the sanctity of Marriage and the dignity of the family’ through promotion of the method, to establish centres to instruct on the method, to assist in further research into the method, and to establish a reference centre incorporating a newsletter.^74^ In April 1979, it was decided that no centre should make a public statement contrary to National Policy, and that all teachers ‘must have charted their own mucus symptoms’.^75^ Evidently, the method placed emphasis on the teachers’ use of their own bodies in order to effectively teach it.

The teachers were also constantly updating their knowledge. For example, at a meeting in March 1978, Mrs Byrne, one of the teachers, gave a talk on the menopause while Mavis Keniry gave a talk on ‘artificial’ means of contraception, ‘this is something all our teachers should be familiar with though not personally, just in case they would be asked questions on them at any time’.^76^ Annual seminars, beginning on a Friday evening and ending on a Sunday afternoon, were also organised by NAOMI, initially in Cork but then in Dublin and other parts of the country including Limerick, Athlone, and Drogheda.^77^ According to Vera, ‘people came from the North of Ireland […] and from Kerry, Cork, Galway, all parts of Ireland there was a representative. I’m not saying it was very strong in all these places but there was a representative from so many parts of Ireland who attended the national meetings in Dublin’.^78^ Vera recalled a seminar in Dublin where Dr. Erik Odeblad, who had conducted earlier research into the link between the change in mucus and fertility, spoke, as well as seminars which included talks by the Billings when they visited Ireland and ‘different gynaecologists who would have come and give a talk in general on fertility. We had Pat Harrison, Mercedes Wilson USA, Prof John Bonnar, Prof de Valera’.^79^ As well as developing their knowledge, the seminars were an important social event for teachers. By the end of the 1970s, Irish Billings teachers had established themselves as leading authorities in this area, while also aiming to professionalise their movement through a system of accreditation, the organisation of seminars, and the creation of an autonomous community. In the following section, I will explore the reasons why women were attracted to the movement.

## The Billings teachers: motivations and backgrounds

As David Geiringer has asserted ‘recovering the agency that ordinary people played in co-producing sexual knowledge is a vital imperative for historicising religious women’.^80^ The Irish women who taught the Billings Method came from a range of personal experiences in relation to fertility. Many were drawn to the Billings Method out of a desire for effective family planning that was natural and in line with Catholic teachings while others were drawn to the method due to issues with conceiving. Lack of access to reliable contraception had a significant impact on women of fertile age for much of the twentieth century. Repeated pregnancies had a physical, emotional, and financial impact on women, while fear of pregnancy created emotional toll and worry and impacted on individuals’ marital relationships.^81^ Niamh, who became a teacher in 1986, in relation to the women who started the service in Cork in the 1970s referred to ‘the lack of any level of contraception. I mean Pat would clearly, she used to talk to me about that like, she said, “I had nothing else”’.^82^ Therese Shorten, the daughter of Noreen Ryan, stated ‘Because up until then, women had no… They had nothing really, I suppose. Maybe the rhythm method and that, but that was not a reliable method. So, it must have been horrendous for women really when you think about it. You were there to procreate and that was your life. Your main role in life. So, very difficult for them’.^83^ In addition, for women who had difficulties in conceiving, there was limited information or support.

Many of the women involved in teaching the method in the 1970s and 1980s had been affected by the marriage bar. Mena, a Dublin Billings teacher, explained that she had worked in the civil service ‘but unfortunately when you left to have your baby you had to leave the civil service. Your marriage bar. So, I don’t know what way my life could have taken because there was very little opportunity to go out and work’. Work in the home meant that involvement in teaching the Billings Method provided an outlet for these women. Mena explained that most of the teachers were ‘more or less the same socio.. you know, yeah, pretty much the same kind of people. Usually women who are at home’.^84^ Noreen worked in a department store in Dublin after marriage explaining, ‘you served your time for three years and then you became an assistant, and left and got married and we didn’t have a great idea about birth control’.^85^ Maura, a teacher in Co. Louth, also recalled ‘college wasn’t really an option for many people because you know, you’d be told well sure well you’re going to get married and have kids and not work and all the rest of it’.^86^ As Deirdre Foley has argued, the marriage bar ‘became the cultural default in many workplaces and subtly but profoundly limited women’s own sense of their horizons’.^87^ For many Billings teachers, involvement in the group thus helped to give them a sense of purpose and an outlet.

Initially, Mavis Keniry and Noreen Ryan began to teach friends and neighbours about the method in Dublin and Cork respectively. Mary had met Mavis Keniry through a group of young Catholic couples called the Thereseians run by a priest called Father Sean Barry which met regularly to discuss natural moral law.^88^ Mary recalled that in 1970 Mavis Keniry ‘invited me to her house to hear the tape that John Billings had sent her, she had got it from Australia, talking about the method. And that was my introduction to the Billings in 1970’.^89^ Vera, who lived in Cork, became involved in the Billings Method in 1971 after having four children between 1964 and 1969. She found out about the Billings Method from her neighbour Noreen Ryan, recalling ‘a few other friends of Noreen’s as well became involved and we all began to chart our cycle. [..] And we found that the symptoms that we should find within our own bodies yes, they were there. We could chart. We could identify them, according to what this talk told us, that the difference between the date when we were possibly fertile and the days when we were not fertile’.^90^

Some oral history respondents were drawn to the method after having difficulties conceiving. For example, Noreen Ryan’s daughter, Therese Shorten, explained to me that her mother became interested in the Billings Method after having fertility issues. She believed that her mother and the other teachers who got involved in Cork did so out of a desire ‘to help women. That there was something there now to help women who didn’t want maybe to have more children or women who wanted to have kids to try and have kids. And I suppose also because the Church recommended it because the Church played a huge role in their lives’.^91^ Shorten’s testimony here highlights the broader aims of both users and promoters of the method in fertility management but also the importance that such fertility management should be in line with Church teachings.

Most interviewees, however, mentioned that they were drawn to the method because it was in line with their Catholic beliefs and/or because of a concern around the side effects of medical methods of contraception such as the pill. Vera was motivated because ‘as a couple we needed a method of family planning. We would not have considered the pill both from a medical and a religious point of view’.^92^ Veronica, who lived in Dublin, found out about the Billings Method through her involvement in the Christian Family Movement in Dublin, and began to train as a Billings teacher in 1978 while she was pregnant with her fourth child. She decided to get involved because the method was natural and in line with the Church’s teaching.^93^ Anne explained that following the birth of three children in 2 years and 9 months and after experiencing post-natal depression, ‘I knew I had to try and do something. For the sake of our marriage, for the sake of my health. And I had…I was mid-thirties. The pill had never appealed to me. But I knew I needed time to breathe, time to, in a sense, recover’.^94^ Anne was put in touch with DOMAS by a priest friend and was taught the method which she found ‘worked and you got guidance all along because you were regularly met by a person who understood what you were learning or trying to do. And they were very supportive. And then I was asked would I like to become a teacher myself and I trained and I did that’.^95^ Anne’s testimony highlights the woman-to-woman element of teaching the method and how women who successfully practised it could then be recruited to teach it.

Some teachers, while Catholic, emphasised that they were drawn to the method primarily because they viewed it as ‘natural’ and involved observation of their own bodies rather than the use of medical or mechanical forms of contraception. Mena came across an article on the Billings Method in a magazine at her mother’s house. Aged 22, after the birth of her first son, she wished to find an effective form of contraception, but due to existing health issues, forms of contraception such as the pill ‘didn’t really suit me at the time because contraception at that stage as you know was very large doses of hormones’. Mena got in touch with Mavis Keniry and was invited to a DOMAS centre in Tallaght.^96^ Noreen also explained ‘I would have always felt in every way that the natural way was best, if at all possible’.^97^ Niamh was introduced to the method by her older sister who brought her to a DOMAS meeting when she was visiting Dublin around 1972. Niamh went on to do a course with her sister in East Africa when she was there from 1974 to 1976. When she returned to Ireland and got married, she and her husband decided to use the Billings Method. She explained: ‘Now I know that many people in NAOMI would have picked Billings because of Catholic reasons. For me, it was natural health reasons, do you follow me?’^98^ Niamh, who had three children, had also been involved in the La Leche League in Cork, an organisation which promoted breastfeeding, and felt that Billings was ‘was kind of a natural follow on, for me’. In 1986, she attended a talk given by John and Lyn Billings when they visited Ireland and at this, was asked by Vera if she would like to train to be a teacher.

Learning about the method had an enormous personal impact for the women I interviewed. Maura, who lived in Drogheda, was introduced to the method by a neighbour, recalling ‘I just couldn’t get over when I did learn you know, about your natural fertility and what you really had there right’.^99^ Mena recalled that it was life-changing for she and her husband ‘because it gave us control which we felt we didn’t have. Do you understand me, at that stage? Two very naïve people now, completely gobeens when it came to anything like this. So, I stayed on, I planned my second baby and then I was invited to become a teacher. So, that’s when I… and I have to say, it totally changed my life. I don’t know what way my life would have gone’.^100^ Many teachers thus became involved in teaching the method because of using it successfully themselves and wanting to spread the information to others. Maura stated ‘because I benefit so much from it, I certainly wanted to go out and you know, give everybody else the great news’.^101^ Eleanor Buckley, a Dublin Billings teacher, in an interview with *Woman’s Way* magazine in 1982, explained that the method had ‘worked very well for us, so well in fact that I was anxious to help others learn about it. I don’t get paid for teaching the Billings Method, so if I didn’t believe it worked, I wouldn’t bother going out to tell others about it’.^102^ Billings Method teachers also needed to be using the method themselves. As Mena explained, ‘Well how can you promote something that you don’t use? Do you know what I mean? You know, you can’t, there’s immediately there’s a doubt’.^103^ The above comments demonstrate the evangelising power of the method.

The women who taught the Billings Method clearly saw their work as apostolic. Perhaps unsurprisingly, the Virgin Mary was a model for understandings of femininity and family life for many of these women who had been born and come of age in the post-war period.^104^ In 1975, the DOMAS minutes reported that ‘Mrs. Keniry thanked ladies for their efforts and said this is apostolic work we are doing and should be under the auspices of Our Lady or maybe the Holy Family. Mrs Byrne of Inchicore convinced ladies it should be under Our Lady of the Immaculate Conception’ while the Rialto teachers decided to ‘put their work under the protection of St. Oliver Plunkett’.^105^ Teacher meetings usually began and ended with some prayers and from September 1977 it was decided to say a decade of the Rosary at the beginning of each meeting.^106^ There was, however, also a tension in that while many of the teachers were Catholic, they felt that the fact the method was associated with the Church was a disadvantage. Maura stated that ‘And I always felt very, very sad that the Billings Method was actually given approval by the Pope and the Catholic Church, because it became known as the Catholic way. Where in actual fact, we’re all looking now at nature and going natural in every way and it’s pity that it wasn’t seen as that rather than you know, the Catholic way’.^107^ It was also common that the method was conflated with the temperature and rhythm methods by members of the general public. Ita believed that ‘they tied it in with that; the rhythm and the temperature and that’s Catholic teaching. And the Billings would have been, they just would have themselves put under that umbrella. [..] We wanted to be inclusive of all women so we were coming from that and whether regardless of what religion they were’.^108^

Ultimately, as this section has demonstrated, women’s reasons for becoming Billings teachers were varied, highlighting the diversity of experiences. All women became teachers after themselves using the method, with many wanting to spread the message to other women who might benefit. Their motivation for teaching the method voluntarily often stemmed from a desire to undertake fulfilling work outside of the home, to form community with other women and in some cases, because they viewed it as apostolic work.

## Transnational connections

The transnational dimensions of the Billings movement are also important to note, although due to the word limits of the article, it is not possible to discuss these in detail. NAOMI teachers were part of a wider international network of teachers and were particularly in correspondence with groups in England and Scotland as well as other parts of Europe which shared knowledge with each other. For example, in 1979, it was reported that Mavis Keniry ‘displayed some teaching charts which are used in Rome. It was decided to order some from Rome when Postal Strike is over’.^109^ The Billings were also in regular correspondence with Irish proponents of the method. In January 1977, Mavis Keniry reported on her meeting with Dr. Lyn Billings in London where she discussed NAOMI’s statement of policy, structure of the organisation, and teacher training, for which she received feedback and suggestions. According to the minutes, ‘Dr Lyn Billing discussed further international bodies and dangers of affiliation to same, who do not uphold the Natural Moral Law. Mrs Keniry said Dublin was not interested in affiliation with anybody not upholding these principals (sic). They also discussed World Health Survey and dangers of same’; the dangers were not expanded on.^110^ The group was also kept updated on developments with the method from the Billings, including subscription to their Bulletin.^111^

In 1978, Mavis Keniry was appointed to the International Committee of W.O.O.M.B. (World Organisation for the Ovulation Method—Billings), based in Los Angeles.^112^ Following a meeting in 1981, Keniry reported to the wider group ‘on some points of her trip to Los Angeles. She brought back some tapes made by K. Hume’.^113^ The Billings also visited Ireland on a few occasions. Dr. Lyn Billings visited Ireland in 1981, a visit that ‘everyone felt that it was very successful and very edifying for all O.M. teachers’.^114^ In June 1985, the Billings conducted a lecture tour of Ireland. In addition to the lectures, they were received by Archbishop McNamara for lunch, met with Bishop Edward Daly for lunch, and met members of the Irish government.^115^ For the Dublin lecture at St. Vincent’s Hospital, invitations were sent to the Catholic hierarchy, politicians, parish priests and clients, as well as pro-life organisations and nurses’ organisations. Posters were put up in churches, chemists, supermarkets, doctors’ surgeries, health centres and hospitals.^116^ It was estimated at the NAOMI AGM that at least 2,000 people had attended the various talks.^117^ In 1987, John Billings wrote to Mavis Keniry updating her on their activities and stating ‘We are very interested to have news of your work. In our travels we are doing what we feel is the most useful thing that we are able to do as a contribution to the family apostolate’.^118^

Catholic nuns were also prominent in circulating information on the method. For example, Sister Maureen O’Sullivan, a member of the Medical Missionaries of Mary in Drogheda, played a significant role in promoting the method there from the late 1970s. Sister Maureen had joined the Medical Missionaries of Mary at the age of 19 in 1939 and trained as a midwife. In 1960, she went to Tanzania, and 5 years later to Ethiopia where she was based for 12 years, returning to Ireland in 1977 where she began to promote and teach the Billings Method.^119^ Eileen, a teacher in Drogheda, first met Sister Maureen when she worked as a nurse in her local hospital in Drogheda, which was run by the Medical Missionaries of Mary. She remembered ‘But she had a whole other life we’ll say in Africa and then when she came home and retired, she got into this family planning and Billings. And as first you were smiling to yourself saying, you know, what are the nuns doing? Family planning?’^120^ Maud recalled ‘we had a lot of meetings of the teachers now, of Billings, we had a lot of meetings with Sister Maureen who always brought us up to date with anything new, or any new developments, or brought us together as a bunch of people’.^121^ Similarly, Jean, who was also taught by Sister Maureen in Drogheda recalled ‘she was a wonderful leader, but she led from behind. She just encouraged everybody else to be their best selves’.^122^ O’Sullivan also produced the newsletter for the NAOMI for many years. Other religious sisters were active in other parts of the country. Sister Ann Healy of the Little Sisters, described in one newspaper as ‘a native of Newquay, now stationed in Dublin’ gave lectures on the method in the late 1970s.^123^ It is unclear, however, if these religious sisters themselves observed changes in their bodies in the same way that lay teachers did.

The group also received correspondence by post and in the early years of the service, they had, according to Vera, ‘a postal system whereby a couple would receive the chart and stamps through the post, they would chart a couple of cycles and send it on and make a phone call’.^124^ In the 1970s, DOMAS regularly received letters from women asking for information about the ovulation method. In November 1975, it was reported at a teachers’ meeting that ‘a lady in Carlow and Irish girl in Spain wrote enquiring about Billings book, chart and stamps sent on, and informed about postal service in Cork’. In August 1976, the group received enquiries from Cork, Dublin, Carlow, and Roscommon.^125^ In September 1977, after an article by a Fr. Nash appeared in the *Sunday Press* about the method, it was noted that ‘there were about 300 queries from England, Ireland and the North and from USA’.^126^ In June 1982: there were 30 enquiries by letter during the month of May: three of these included orders for a book on the method.^127^ This again demonstrates the dearth of basic information available in Ireland at this time around fertility control. To address this lack of information, several guides on natural family planning methods were also published from the 1970s by NAOMI. One such guide, *Natural Family Planning: Ovulation Method (Billing)* was produced by the Ovulation Method Advisory Service in Cork, affiliated to the national association.^56^ The guide contained a foreword by Rev. Dr. Lucey, Bishop of Cork and Ross, which emphasised the importance of responsible family planning and the expectation that marriage should produce children. Readers could send for a chart and stamps from the NAOMI address. However, it was generally agreed that the best way for the method to be taught was in person by another woman; this reasserted the teachers’ sphere of authority in this area.

## Teaching the Billings Method

The experiences that teachers had with women who came to the centres reveals much about the wider climate in relation to family planning and the impact of the lack of sex education in the country. In the 1970s and 1980s most clients were trying to postpone a pregnancy with the minority trying to achieve a pregnancy. Speaking about the clients, Eileen recalled ‘I’m not sure they even enjoyed their sexual side of their life because there was such a fear of becoming pregnant for a lot of people’.^128^ As research by Máire Leane on Irish women’s sexual experiences has shown, these were often accompanied by fear and anxiety, with women’s narratives demonstrating ‘how difficult it was for women to assert a sexual persona that did not comply with prevailing normals and practices’.^129^ Moreover, there was a significant absence of a discourse on sexual pleasure in Ireland in this period. Within *Humanae Vitae*, and in the correspondence it generated, sex was primarily established as ‘fundamentally procreative’.^130^

Due to a lack of formal sex education in Ireland and the stigma around reproductive health matters, many women knew very little about their fertility or reproduction. Ita recalled ‘in the early stages, like women hadn’t a clue how their bodies were working, you know, and men particularly hadn’t got a clue’. The Billings Method also gave individuals ‘the vocabulary as well, because a lot of people wouldn’t, they’d be shy about things and it would never been covered in schools or their mothers mightn’t even have spoken to them before they got married or anything. So, there was a lot of fear and a lack of knowledge about what actually is going on’.^131^ Given the sensitive nature of discussions, some women went to a centre that was outside of their own locality. Mary explained ‘Because the people wanting to be private didn’t want to be seen by a neighbour, that “Oh, she knows why I’m here, I know her business,” type of thing. So, even people would drive distances to go to a centre so that nobody might see them’.^132^

According to teachers, clients came from a range of backgrounds, depending on the location of the centre. As Ita, who volunteered primarily in the centres on the north side of Dublin, explained, ‘Now, some of the areas were what you would call poorer areas. And then you’d get the middle-class areas. Very rarely upper class to be honest with you, they’d be a kind of a mixed bag’. Typical clients were ‘stay at home mothers, husband out all day, she’s got you know, four/five children at home, doesn’t want anymore or she might want another one but she doesn’t want it immediately, you know. [..] And they would have known very little about their fertility, wouldn’t even want to talk about it’.^133^ Noreen taught in Ballymun, a disadvantaged part of Dublin, explaining ‘some parts of it are very deprived. People piled into flats without anything around them, and yet very big housing areas of private houses. It was a very big mix’.^134^ At a meeting in September 1975, a representative of the Ballymun centre reported ‘satisfactory progress was reported but the absence of clients from the Ballymun flats is very remarkable. Most of those who come are from nearby Collins Ave, and Finglas area’.^135^ Anne stated that in the 1970s and 1980s, ‘I would say a lot of people were what we would call from ordinary backgrounds. Now most of us, including myself, are from very ordinary backgrounds. But yes, we did meet people who were in professions and again, the penny dropped quickly and they got it and were very happy. But Joe and Mary Average would have been the bread and butter of the clientele’.^136^ In Drogheda in the 1980s, Eileen believed that the women coming to the centres there ‘were all pretty middle class, that they’d heard it from a friend of a friend. There were very few that … of the lower socioeconomic groups’.^137^

The history of the Billings Method is also revealing in terms of understandings of gender in the late twentieth century. There was an implicitly feminist ethos and appeal to the Billings Method and a sense that having knowledge of fertility could be empowering. For example, an extract from the *Billings Atlas* in 1989 stated: ‘Self awareness of fertility and infertility is important knowledge which should be available to every woman […] Many benefits accrue, including the ability of the woman who knows her own mucus pattern in times of health to detect early indications of a number of gynaecological disorders’.^138^ Jane Quinlan, of the Family Life Centre (Billings) in Cork, writing in response to a critique of the method in the *Irish Times* in 1981, argued that ‘the Billings Method has to do with education (*educere*—to lead out of ignorance) on Reproduction. As such it has to do with the true liberation of women’.^139^ Similarly, Dr. Esther Bradley, the medical director of Catholic Marriage Advisory Council (CMAC), in a newspaper article in 1981, suggested that natural family planning methods had ‘aesthetic, health, spiritual, even feminist appeal’.^140^ Yet, given that the key goal of the method was the practice of family planning in line with Church teachings, rather than offering women the option of a range of choices, it cannot be viewed as feminist, and teachers themselves would not have described themselves as such.

On a personal level, Billings teachers found that the knowledge of their cycle was empowering. Sarah stated ‘it’s great that you’re in control of your own body you know, you can see what’s happening, you know’.^141^ Similarly, Jean explained:

It was great to have it for when people did try the pill for five years and decide to come off it. We were there to guide them back and encourage them. So, as that when they did see ovulation, even if it was only every third month or whatever, that they actually knew what was happening. So, that empowered them, and it saved them a lot of expense. […] So, even knowing that was wonderful, that knowledge was wonderful, and the woman then actually doesn’t need any doctor, any nurse, any test to tell her; she has that herself.^142^

As Jean highlights here, the method empowered women with knowledge of their cycles, avoided the need for medical supervision and was economically beneficial due to the lack of cost involved.^143^

The group also shared an anti-medicalisation stance with feminist health campaigners. In a 1979 newspaper article, Mavis Keniry commented that many women who came to the Billings centres ‘approached their doctors and this [the pill] was all they were offered, which I think is a sad situation’.^144^ Niamh shared this perspective, explaining ‘Because if that same person goes to their doctor, no offence, but the doctor will be so happy to prescribe medicine, you know what I mean? Whereas you’ll never be told by your doctor “Well, have you tried this?” Yeah, so it is women to women you know’.^145^ Mary Kennedy, an anti-contraception campaigner and member of the Irish Family League, wrote to the press in 1976 to say ‘We have been told by women seeking information on natural methods: “He would not spend five minutes to discuss the problem with me, but just wanted to write a prescription for the Pill.” Other women have told us that doctors did their best to persuade them to take the Pill when they went for a post-natal check-up, even though these women had not asked for advice and were indeed already adequately spacing their families’.^146^

Promoters of the Billings Method were also concerned with the side effects of the contraceptive pill which was prescribed by Irish doctors for women with irregular cycles in the period before the legalisation of contraception. As Lara Marks has shown, members of the medical profession in the 1970s and 1980s in the UK and USA vigorously debated the side effects of the pill.^147^ The links between the pill and thrombotic disease were particularly of concern in the wake of the thalidomide tragedy.^148^ In the USA and the UK, the perceived over-prescription of the contraceptive pill was heavily critiqued by feminist activists, particularly in the wake of Barbara Seaman’s 1969 book *The Doctor’s Case Against the Pill*.^149^ These side effects were also well-documented in Ireland. An article in *Woman’s Way* in 1979 drew attention to the experiences of a number of women who had side effects on the pill including depression, lack of energy and lack of sex drive which made them decide to come off the pill and try and different form of contraception.^150^ In a letter to the *Evening Press* in May 1979, Mavis Keniry highlighted that in Britain, ‘a contraceptive pill victim action group, with a membership of over 300 has been established. Complaints range from thrombosis, strokes, infertility and death. The aim of this organisation is to sue for compensation for those suffering illness or loss, and to make the public aware of the effects of such medication as prescribed by doctors’.^151^ Radical feminist groups such as Irishwomen United and the Contraception Action Programme were similarly critical of the pill, which was prescribed as a cycle regulator to Irish women prior to the legalisation of contraception in 1979. In 1976, Irishwomen United stated, ‘We demand the BEST and SAFEST forms of contraceptives FREE. Women are not guinea-pigs. We don’t want to have to put up with expensive contraceptives that either don’t work or make us feel ill or depressed’.^152^ Feminist activists were often critical of the medical profession for not discussing a full range of contraceptive options with women.

Such sentiments were shared by advocates of the Billings Method in Ireland. Sonya Keniry, Mavis’ daughter, felt

I started to realise very early on that the people on the other side, they were all fighting the same cause, but as far as my mother was concerned, their information was limited, and she absolutely felt that they were being sold a pup, she really did. There was no… she definitely did, she felt that women were being commodified, that everything to do with women, they had to buy, they had to purchase, it was going to be marketed to them, and this really incensed her, like, really incensed her. She felt it was a further abuse of women, I suppose, that the knowledge was out there and it wasn’t being shared with them.^153^

A 1973 article in the *Evening Herald* included the testimony of a woman who used the method who stated, ‘We, women, are fed up with people pushing pills at us. I discovered the Billings Method and it is my answer to the pill. I wish more women knew about it’.^154^ Deirdre Manifold, also a campaigner against contraception, established a Billings centre in Galway in spring 1976.^155^ Writing to the *Connacht Tribune* in 1977, she outlined the benefits of the method, including that ‘There can be no strain from fear of side effects of pills or I.E.D. which do pose a real danger’. Quoting Herbert Ratner who had stated ‘Women make superb guinea-pigs- they don’t cost anything, feed themselves, clean their own “cages” and pay for their own pills’, Manifold argued ‘No sensible woman wants to be a guinea-pig and it is only because vital information is being kept from her that she is so treated’.^156^

However, there were significant differences between the approach of proponents of the Billings Method and feminist health activists. An important difference to note was the ‘pro-life’ stance of the Billings Method. John and Lyn Billings, in their *Billings Atlas of the Ovulation Method*, stated that teachers should exhibit a broad range of personal qualities as well as a pro-life ideological positioning. The Billings suggested that as well as having ‘an adequate knowledge of the method, the ability to impart confidence and to dispel anxiety’, as well as traits such as patience and the ability to impart knowledge in an accessible manner, teachers should exhibit ‘compassion and friendliness based on respect for human life and human dignity’ and should have the ability to communicate to the couple an attitude of total acceptance of every child.^157^

The link between the Billings Method and ‘pro-life’ teachings is clear here in the discussion of the ‘respect for human life’, and ‘an attitude of total acceptance of every child’. Similarly, as Agata Ignaciuk’s study of the work of Polish gynaecologist Włodzimierz Fijałkowski, a proponent of natural family planning and the creator of Polish childbirth schools in post-war Poland, has shown his strong links between natural family planning and a pro-life ideology.^158^ This outlook evidently contrasts with feminist health activism, of which the key tenets were choice, women’s agency, and access to abortion and contraception. Yet, it is evident that promoters of the Billings Method publicised it using the some of the key tropes of feminist health activism, while also adhering to twentieth-century Catholic theological teachings in relation to sexuality and family planning. And, in line with Martina Avanza’s recent scholarship on female pro-life activists in Italy, scholars should avoid ‘falling into normative judgements (are they feminist or not?) that would diminish our capacity to fully understand conservatism’.^159^ As will be discussed later, it is unsurprising, however, that many Irish Billings teachers would go on to be involved in the PLAC (1981–3).

The Billings Method also meant that responsibility for family planning was shared between both partners, thus drawing on ideas promoted in *Humanae Vitae* around ‘companionate marriage’ and ‘mutuality’.^160^ In an interview with journalist Mary Leland in 1973, Cork teacher Noreen Ryan commented, ‘It is best taught by women to women but it makes sense to the husbands too because it is something that has always been in nature. The wife is personally involved, but the husband can ask very searching questions, and they say that using this method has made a great difference to their marriage in terms of togetherness—there’s a sense of working their difficulty out together’.^161^ Such ideas were also proliferated by Catholic lay activists in organisations such as the CMAC who characterised ‘true married modern love as biblical—patient and kind, non self-seeking and ever-preserving’.^162^

Because the method required abstinence from sexual intercourse on certain days of the cycle, it required cooperation from the wife’s husband. As Natalia Jarska and Agata Ignaciuk have recently shown in the case of fertility control in state-socialist Poland, ‘Power relations in marriage were, therefore, of crucial importance in reproductive decision-making’.^163^ Mary explained ‘even though men are painted in such a way in that “Oh, you couldn’t ask your husband to go by abstaining during those few days.” But I mean why not? Men are able to control their sexuality, because sexuality is not just the physical act of intercourse. Sexuality, to me, is all around you. It should be through your day, through the way you talk to each other, through the way you behave with each other’.^164^ Mary’s comments here demonstrate understandings of male sexuality as outlined in *Humanae Vitae*. As Alana Harris has argued, the encyclical portrayed masculinity and male sexual desire as ‘lustful, insatiable and even bestial. The pill, through this optic, would infantilise Catholic husbands, blinding them to their paternal responsibilities and inhibiting moral growth and spiritual maturity. Readily “indulged” without the natural inhibitor of fear of pregnancy and impending breadwinner responsibility, male sexuality had the potential to distort, to corrupt and even to kill love’.^165^ Catholic family planning activists in other countries also echoed such views. In Polish Catholic family planning literature from the 1930s, it was suggested that ‘periodic abstinence using the “calendar” method was therefore also intended to protect women from male sexual appetites’.^166^ And, from the 1980s, Włodzimierz Fijałkowski, a key promoter of natural family planning in Poland encouraged men to educate themselves about the female fertility cycle and that ‘cycle-observation should be a calendar to guide a man’s life and fatherhood’. More broadly, this was situated within his vision of a more equitable marital relationship.^167^ Such ideas were promoted in the teachings of the Billings Method. For example, when an engaged woman wrote to Irish agony aunt Angela Macnamara in 1974 for advice on family planning methods, Macnamara advocated the Billings Method, stating: ‘There is nothing very refined or dignified about killing off a husband’s sperm or disrupting a normal course of nature in the wife’s uterus. I hope you two will get together and learn this aspect of loving which certainly calls for control, but you’ll find that it is good for both of you and for the quality of your marriage’.^168^

## Tensions and resistance

The women involved in teaching the Billings Method also actively defended it, particularly against accusations that it was not effective, thus positioning themselves as authorities in this area. ‘User failure’ was generally deemed to be the cause of the method not working. In his 1976 reference paper ‘Evaluating reports of Ovulation method effectiveness’, Rev. Dermot Hurley wrote that user failure occurred because ‘Clients sometimes fail to learn the method, or fail to learn it well, because of lack of motivation. They do not really believe that it is worth learning!’ He further stated that ‘When a client gets pregnant unexpectedly, the instructress will, if she can, try to find out why. Often after a few questions, the pregnancy can be classed as a “user failure,” and efforts made to improve the motivation of the couple, and their understanding of the method’.^169^ Here, Hurley was suggesting that failure of the method was usually down to a lack of motivation or lack of knowledge on the part of the couple.

In 1979, the DOMAS minutes record that a letter was sent to RTE regarding ‘a remark made on the programme “Women Today” that O.M. didn’t work with menopausal women. In the letter K. O’Toole stated that O.M. [Ovulation Method] is very effective for women going through the Menopause and she included an address and phone no. to be broadcast where further information about OM can be obtained’.^170^ Gay Byrne, a popular Irish TV and radio host, was written to in 1980 ‘about Dr’s comments on O.M. on one of his programmes. Letter corrected some of the erroneous remarks he made and suggested if they were discussing O.M. in future on Radio to contact the experts!’^171^ And, in 1981, Mavis Keniry publicly critiqued the Late Late Show for not including Dr. Lyn Billings, who was visiting Ireland at the time, in an episode of the programme. At a press conference, Keniry stated that promoters of other forms of family planning had appeared on the show twice recently, and that ‘Thousands of Irish women are interested in natural family planning methods’.^172^ Ita, believed that this incident revealed that ‘there was a huge bias there’ against the method.^173^

There was some resistance from members of the medical profession to supporting the method. In 1975, it was reported that the doctor of a woman attending a centre in Donaghmede that ‘had [inaccurately] told one of this patients that it would not be possible for her to use the Billings Method while she was breastfeeding’.^174^ The same year, it was reported at a meeting ‘that Dr. Gallagher, Ennis, made a statement that the Ovulation Method doesn’t work for some women. Mrs. O’Toole is writing to clarify this’.^175^ In 1976, ‘It was decided to enlist the help of doctors in each area with ovulation method after Easter at least to request them not to condemn the method’.^176^ And, in 1981, when NAOMI established a centre at the Mater Hospital, they reported ‘The O.M. teachers have been getting a feeling that the Hosp. want to run the Centre themselves. [..]. Mavis thinks that getting Drs run the Centre would not be the best thing for prom. of method. The clients are inclined to be treated as patients not clients, also one of the nurses appears to be teaching three methods.^'177^ Similarly, when Professor John Bonnar was awarded a grant of £17,500 by the World Health Organization to undertake a pilot study of the Billings Method, as part of a five-country survey, there was some wariness among members of the DOMAS executive about taking part. In February 1977, at a DOMAS meeting ‘Mrs G Keogh read out warning given in latest Bulletin of NFP Council of Victoria, it was stated in same that in past WHO Surveys clients taking part were advised to use barrier methods of contraceptives during fertile days. It was the feeling of all teachers present that before we take part in proposed WHO Survey that more information on the way it is to be conducted be available to us’.^178^ This illustrates the tensions around combining natural forms of family planning with other methods against the more restrictive approach of the Billings Method. Following a seminar organised by Bonnar and Dr E. Bradley, teachers appear to have had their concerns allayed but just three of those attending volunteered to teach the method.^179^ Yet, despite these tensions, NAOMI usually had a senior male member of the medical profession act as their president. In 1979, this was Dublin gynaecologist Arthur Barry.^180^ As with the PLAC in the 1980s, the representation of senior members of the medical profession may have helped to legitimise the women’s activism.

In some ways, the work of Irish Billings teachers has parallels with the work of the CMAC in Britain. In relation to CMAC, Alana Harris has argued that ‘in this work, these middle-class and well-educated Catholics were indeed usurping something of the role (and authority) of the parish priest, through effectively hearing personal “confessions” and effecting “reconciliation” in key areas relating to family life and morality’. This meant that the CMAC ‘had to assert its unique and “modern” contribution in this area bolstered by emergent social scientific knowledge, while at the same time observing lay-clergy hierarches and skirting doctrinal controversies’.^181^ It is evident that support from the Church hierarchy in Ireland for NAOMI was mixed. In the 1970s, the group had limited financial resources and regularly contacted local priests and bishops for financial support. In August 1976, DOMAS reported that they had ‘£5 odd in the red!’^182^ In September 1976, it was reported in the minutes that Mrs. O’Toole, one of the teachers, had written to 12 priests ‘looking for money on August 23rd—no replies received’ while the group also wrote to archbishop of Dublin, Dermot Ryan, ‘asking him for finance’.^183^ By February 1978, finances had improved with the treasurer Mrs. G. Keogh reporting a financial statement of £432.^184^ Occasionally, DOMAS received donations from local priests, for example in May 1979 when they received a donation of £70 from a Father Gallagher in Tallaght.^185^ However, the group spent a lot of time writing to request donations. For example, in May 1977, Mavis Keniry and Kathleen O’Toole met with Archbishop of Dublin, Dermot Ryan, where they ‘stressed the need for finance and covered various other subjects including teacher training, postponed seminar, achieving publicity, literature given to clients, lists given to various hospitals’.^186^ And in November 1979, the minutes record that ‘the critical financial situation was then discussed’. A letter was sent to Irish based firms ‘appealing for finance to enable O.M. to continue. It was to be a once only appeal. […] A letter is also to be sent to the four Archbishops requesting fund. No reply has been rec. to last letter’.^187^ In 1979, after receiving a donation from Cardinal O’Fiach, the group wrote to thank him and stating ‘the lack of support we were receiving in some dioceses & parishes and the need to vigorously promote this method of F.P.’^188^

Some local priests did provide support in the form of small donations or the use of parish spaces to run centres. Mena felt that ‘They weren’t ready, they were too staid in their ways, they didn’t want to get involved in case they had, they recommended us to couples and the couples came back and said they were pregnant. They weren’t prepared to take that chance. You know that kind of a way. They supported us in little ways like giving us rooms or they’d support us, you might get a contribution.’^189^ In 1981, for example, it was reported in the DOMAS minutes that Kathleen O’Toole ‘made 97 calls to various parish priests or priests who represented their parishes at the deanery meetings. She reported a mixed reaction.’^190^ In 1986, a letter was sent to all parish priests in Dublin to highlight the work of the organisation, and explain that they were ‘renewing our efforts as we are reminded of the apostolate of the laity, and are requesting your co-operation in the promotion of same. […] As you are aware, the family is under increasing attack, and we are renewing our efforts to stem the tide—therefore we need your help’.^191^

There were also some tensions between the organisation and the CMAC when NAOMI began to organise pre-marriage courses from 1980; these were held for engaged couples before they got married in a Catholic church.^192^ The first pre-marriage course was held in autumn 1980 by Ita McDonnell and Mavis Keniry at Killester Technical School. In April 1984, one of the Billings teachers, Margaret, reported that the pre-marriage course she had been involved in ‘was the most worthwhile project she has ever undertaken. She also mentioned that CMAC were objecting to her course and she felt it might be a bit difficult organising a further course. She hopes to talk to her local Bishop’.^193^ Ita recalled

the church would support their own courses. So, we were kind of on the fringe of all of that. They never quite; they never… We would have had a list of priests that would always send their couples to us but it wasn’t, we weren’t the mainstream. And they still would have been a little bit scared I think of natural family planning, of the Billings.^194^

As Cara Delay has argued for the earlier period, ‘despite an intensified Church-led Catholic patriarchal culture, lay women did what they could from 1850 to 1950 to maintain autonomy and influence their faith’.^195^ Likewise, the women involved in DOMAS were also unafraid to request more leadership from the Church hierarchy in relation to the issue of family planning. The group wrote to Archbishop of Dublin Dermot Ryan in 1979 to complain about remarks made by moral theologian Fr. Maurice Reidy on the Women Today programme where he apparently stated that women had ‘the right to choose other methods of F. Planning as N.F.P. does not suit all people and a lot of research needs to be done. Question asked of the Archbishop—have women the right to choose contraceptives and birth control methods’.^196^ In March 1980, it was reported in the minutes that ‘Mrs. Keniry had a meeting with Archbishop’s Secretary. She criticised Fr. Reidy’s remarks on the “Women Today” programme, and would like His Grace to put down on paper the Church’s teaching. She also said it was time the O.M. Organisation got the recognition it deserved’.^197^ And, in May 1980, a letter was sent to ‘Bishop’s Sec. requesting the Church’s teaching in writing’.^198^ The group do not appear to have received a reply on this matter. In December 1988, the Diocesan Council for the Family, led by the Archbishop of Dublin, forwarded Keniry a cheque for £2,000 to assist in their work, specifying that ‘this donation is not, nor can it be an annual grant’.^199^ Ultimately, as this section has shown, the Billings Method enabled teachers to carve out an area of authority and Catholic activism, yet the Church hierarchy's response was lukewarm, emphasising a wariness of lay movements, particularly female-led ones, and fear of endorsing a family planning method that might fail.

## Future activism

The teachers also had wider concerns about changes in society and kept informed about the work of more left-leaning groups, sometimes even attending their meetings. For example, in April 1975, two teachers, Mrs. O’Connor and Mrs. Cassin reported on a seminar held by the IFPA in Mountjoy Square by the IFPA.^200^ In November 1975, Mavis Keniry reported on a meeting held at Liberty Hall by Irishwomen United.^201^ And in November 1976, the group’s minutes reported that Mavis Keniry had noted that there ‘is a group in 21 Templeogue Rd calling themselves C.A.P. Contraception Action Protection (sic) who are campaigning for legalisation of Contraceptives.’^202^

The women were at times, more alert to perceived threats from the left, than the Catholic hierarchy. In January 1989, Mavis Keniry wrote to the Archbishop of Dublin, Desmond Connell, to inform him that ‘in the new syllabus for Science in the Junior Certificate Course, due to commence in September 1989, the topic of “contraception” is to be introduced. This will apply to children from the ages of 11 to 15 approximately. We feel that this is a grave infringement of the rights of parents and their children’.^203^

It is, unsurprising that many, but not all, Billings teachers got involved in the abortion debate which emerged in 1970s Ireland. Veronica, who campaigned against abortion and divorce in the 1980s, saw her involvement in these campaigns as a logical follow-on from her work promoting the Billings Method, stating ‘it is all tied into natural family planning. As in, having respect for the natural method’.^204^ DOMAS was in correspondence with international anti-abortion campaigners. The minutes also recorded in 1976 that American activists Dr. Kevin Hume, Father Paul Marx, and Herbert Ratner, were coming to Ireland in September and discussed the possibility of organising a talk by them.^205^ In September 1976, the teachers’ group organised a meeting with Dr Hume at the North Star Hotel Dublin while a public lecture in the national stadium with Dr Hume and Dr Marx in Sptember was also noted in the minutes.^206^ DOMAS was also in correspondence with Peggy Norris, an Irish SPUC campaigner who lived in Liverpool.^207^ Norris wrote to DOMAS in June 1980 about a Pro-Life Conference which was to be held in Dublin that September, and at the DOMAS meeting, ‘it was suggested that O.M. Teachers attend some of the talks’.^208^ And, in 1982, Mavis Keniry reminded teachers of ‘the “American night” on the 18th March where Fr. Cochran, and Mr. Pat Trueman would be the guest speakers. There would also be refreshments’.^209^

Fourteen anti-abortion groups mobilised in 1981 under the banner of the PLAC with the aim of having an amendment included in the Irish Constitution which would guarantee the right to life of the unborn child from the moment of conception; NAOMI was one of these groups.^210^ As Chrystel Hug has argued, ‘the referendum campaign was the longest, the most virulent and the most polarised there ever was’.^211^ For members of the PLAC, ‘the issue was much wider than abortion since it was about the social and moral future of Ireland: the old values were in danger and were already somewhat eroded by 1983’.^212^ At the DOMAS Executive meeting in February 1981, it was reported that two DOMAS members, Kathleen O’Toole and Mary Doyle had attended a meeting at Mount Carmel ‘as representatives of N.A.O.M.I.’ and that ‘a committee was formed at this meeting on 24th Jan ‘81. M.Doyle be part of this committee. Next meeting Mount Carmel on 7/2/81’.^213^ PLAC successfully campaigned for a referendum in 1983 regarding the introduction of the eighth amendment to the Irish constitution which enshrined protection for the unborn into the Irish constitution and effectively outlawed abortion in the country.^214^ In the early 1980s, reports on the PLAC campaign were given at meetings of the executive. For example, in June 1981, Mary Doyle, a DOMAS member, gave a progress report on the campaign to date.^215^ There appear to have been some disagreements among members of NAOMI in terms of how the PLAC was being run. In June 1981, it was remarked in the minutes that ‘Mavis [Keniry] feels the campaign is not aggressive enough’.^216^ And, the following month, at a DOMAS Executive Meeting, a discussion was held on the PLAC and it was agreed that a letter should be sent to the secretary of PLAC to ask:

1. What were the projected plans for 1981/82 for the achievement of the aim of the Pro-Life Amendment of the Constitution Group?
2. What had the group organised internally to co-ordinate the efforts of all associated organisations?
3. What had the group organised externally, e.g., education of the public as to the necessity for the amendment to the Constitution?
4. What was the costing of a public campaign?
5. What was the financial target?
6. How did the group hope to achieve this target?

This letter was to be signed by Mavis Keniry in her capacity of P.R.O. for N.A.O.M.I.^217^ This letter suggests that there were some frustrations among members of NAOMI in relation to the organisation of the PLAC and that the group wanted clarification on the campaign plans. It appears from the minutes of DOMAS that NAOMI members were active in the campaign, but not in positions of authority. Nevertheless, members of NAOMI acted as footsoldiers, and engaged in door-to-door canvassing as well as, in one member’s experience, the holding of small meetings in women’s houses in order to show anti-abortion slides and films. Moving into 1982, updates on the PLAC continued to be discussed at regular DOMAS meetings. For example, in March 1982, Mary Doyle reported that ‘The people of PLAC had hoped for a meeting with Mr. Haughey, who has promised an amendment to the Constitution, with the Attorney General present.^'218^ In September 1982, Mavis Keniry reported on the PLAC National Co-ordinators Meeting, informing the group that ‘A question and answer booklet has been completed and is to be published shortly. It is hoped that the Bill for the Referendum will be introduced in the Dail during Oct/Nov.'.^219^ And, in March 1983, it was reported that ‘P.L.A.C. are holding no more meetings at the moment. The campaign has been taken over at this stage by the media. It is hoped that the correct wording for the referendum will be sorted out very soon’.^220^ The referendum on inserting the eighth amendment into the Irish constitution occurred on 7 September 1983. The turnout was 53.67 per cent, and the people voted to insert the eighth amendment into the constitution by a majority of 66.9 to 33.1 per cent. At this point in time, it appeared that conservative values had won the battle against rising liberalism in relation to questions of sexual morality, and in the 1980s, anti-abortion groups consolidated their position through a series of High Court injunctions against groups which were providing information on abortion.

## Conclusion

In 1978, the Irish government moved to pass legislation to legalise contraception under the Family Planning Bill. That year, DOMAS wrote to then Minister for Health Charles Haughey ‘seeking appointment to make method more widely available’ and Mavis Keniry met with Haughey to try to obtain State support for the method.^221^ Members were opposed to the legalisation of contraception and the proposed bill.^222^ While the Family Planning Act legalised contraception, it also resulted in a grant for groups which promoted natural methods of family planning, meaning that from the 1980s, the financial situation of the group improved. In February 1981, the group received their first grant of £35,000.^223^ In July that year, DOMAS purchased an office at 16 North Great George’s Street with a part-time secretary attached, while another office was set up in Cork.^224^ The organisation became more professionalised, purchasing a second-hand projector, encouraging standardised equipment at all centres, and from 1984, teachers were given certificates as proof of qualification and were encouraged to wear a teacher’s badge at all centres.^225^ While the funding was beneficial for the organisation, by the late 1980s, numbers of clients were beginning to decrease. At the 1989 NAOMI AGM, it was noted that ‘reports given at the Seminar showed a falling in numbers of new clients attending centres in some places’.^226^ Veronica felt that while in the 1970s ‘there used to be queues outside the doors of the centres but after the Family Planning Act in 1979, the numbers started falling off dramatically. And unfortunately, I think when people think that something is legal, they think it is morally okay as well’.^227^ Maura recalled ‘Now I stopped teaching the method mainly, sadly, because people stopped coming. And I think that probably contraception just became more available and I can absolutely, I feel very sad about this now’.^228^ Several teachers also acknowledged that the method’s continued association with the Catholic Church, which they themselves sustained, affected its popularity in this period in the wake of scandals in the Church. Into the 1990s, many centres closed, however, the Cork and Dublin offices remained open and tended to receive queries from women who were trying to conceive.

For the women who volunteered as teachers, the work had a huge personal and social impact. Women developed life-long friendships because of the work. For others, as well as being rewarding, involvement in NAOMI helped to boost their confidence and self-esteem. Women, both teachers and users alike, were drawn to the method primarily because it was natural and in line with Catholic Church teachings. While the method declined in use in the 1980s and 1990s, during the 1970s, the method provided many women with a means of family planning in a period when there was little else available. Indeed, as Laury Oaks has suggested in her recent article on feminist fertility awareness in the post-Roe world, ‘fertility awareness presents an inclusive, anti-racist, anti-classist, all-bodies, sexual, and reproductive politics and practice that reflects the foundations of reproductive justice’, addressing ‘barriers to contraceptive access, decentres provider-controlled methods, and responds to questions about the side effects of hormonal methods’.^229^ While the women who were involved in promoting the Billings Method in Ireland adhered to the Catholic ethos in relation to family planning and respect for life played a significant role in shaping their work, it is far too simplistic to view their work as simply Catholic or conservative activism. This article has instead argued for a more nuanced view of Catholic women’s activism. Rather than seeing a binary of conservative (Church) vs progressive (society), this complicates understandings of Catholic action in this period. It has shown how the work of Billings teachers, through its encouragement of woman-to-woman teaching, emphasis on women’s empowerment through knowledge, and rejection of medical methods due to both their side effects and religious reasons, may be viewed as bridging both Catholic action and feminist health activism, yet also weaved together elements of twentieth-century Catholic theology and understandings of the body, sexuality and marriage. While the legacy of this movement is difficult to ascertain, it is evident that at a tumultuous point in Irish social history, it offered many women a means of fulfilling lay apostolic work, and for some, an entry-point into anti-abortion activism.

## Footnotes

1 E. L. Billings et al., ‘Symptoms and Hormonal Changes Accompanying Ovulation’, *Lancet*, (1972), 282–84, on 283-4.

2 Mary Leland, ‘Billings Comes to Cork’, *Irish Times*, January 22, 1973, 12.

3 Fr. Dermot Hurley, *Billings Ovulation Method: A Pastoral Approach for Priests and Other Parish Workers* (London: Catholic Truth Society, 1994), 5. [Mavis Keniry Papers].

4 ‘Billings method – a blessing from heaven?’, *Evening Herald*, December 14, 1973, 37.

5 Mary Leland, ‘Billings Comes to Cork’, *Irish Times*, January 22, 1973, 12.

6 ‘Why the Billings method is best’, *Sunday Independent*, March 26, 1978, 14.

7 Alana Harris, ed, *The Oxford History of British and Irish Catholicism*, *Volume V: Recapturing the Apostolate of the Laity, 1914–2021* (Oxford University Press, 2023), 1–11, on 1.

8 Harris, ‘Introduction’, p.2.

9 Lucia Pozzi, *The Catholic Church and Modern Sexual Knowledge, 1850–1950* (Palgrave Macmillan, 2021), 196–209.

10 See: Pozzi, 219–47.

11 Alana Harris, ‘Reframing the ‘Laws of Life’: Catholic Doctors, Natural Law and the Evolution of Catholic Sexology in Interwar Britain’, *Contemporary British History*, 34 (2020), 529–54, on 543.

12 Harris, ‘Reframing the ‘Laws of Life’’, 543.

13 Alana Harris, ‘Introduction: The Summer of ’68 – Beyond the Secularization Thesis’, in Alana Harris, ed, *The Schism of ’68: Catholicism, Contraception and Humanae Vitae in Europe, 1945–1975* (Basingstoke: Palgrave Macmillan, 2018), 1–22, on 7.

14 Laura Kelly, *Contraception and Modern Ireland: A Social History, c.1922-92,* (Cambridge University Press), 151–83.

15 Kelly, *Contraception and Modern Ireland*, 101–104.

16 David Geiringer, ‘Catholic Understandings of Female Sexuality in 1960s Britain’, *Twentieth Century British History*, 28 (2017), 209–38, on 236.

17 Pope Paul VI, *Humanae Vitae: on the regulation of births*, (1968), accessed via: https://www.papalencyclicals.net/paul06/p6humana.htm

18 Alana Harris, ‘A Magna Carta for Marriage: Love, Catholic Masculinities and the Humanae Vitae Contraception Crisis in Britain’, *Cultural and Social History*, 17 (2020), 407–29, on 412 and 423.

19 Avril Baigent et al., ‘Lived Catholicism: A Roundtable Discussion’, *Ecclesial Practices,* 9 (2022), 9–27.

20 The history of ‘natural’ methods is discussed in Donna J. Drucker, *Contraception: A Concise History* (MIT Press, 2020).

21 See, for example: Agata Ignaciuk and Agnieszka Kościańska, ‘Regendering Childbirth: Catholicism, Medical Activism, and Birth Preparation in Post-War Poland’, *Journal of the History of Medicine and Allied Sciences*, 78 (2023), 249–69; Natalia Jarska and Sylwia Kuzma-Markowska, ‘Explaining the Calendar: the Catholic Church and Family Planning in Poland, 1930–1957’, *The Historical Journal,* 666–88 and Sylwia Kuzma-Markowska and Agata Ignaciuk, ‘Family Planning Advice in State-Socialist Poland, 1950s–80s: Local and Transnational Exchanges’, *Medical History*, 64 (2020), 240–66.

22 See, for example: Harris, *The Schism of ’68;* Kelly, *Contraception and Modern Ireland*; David Geiringer, *The Pope and the Pill: Sex, Catholicism and Women in Post-War England* (Manchester University Press, 2019); Caroline Rusterholz, ‘Reproductive Behavior and Contraceptive Practices in Comparative Perspective, Switzerland (1955–1970)’, *The History of the Family*, 20 (2015), 41–68 and *‘Deux enfants c’est déjà pas mal’, Famille et fécondité en Suisse, 1955–1970* (Lausanne: Editions Antipodes, 2017); Leslie Tentler, *Catholics and Contraception: An American History* (Cornell University Press, 2004); Diane Gervais and Danielle Gauvreau, ‘Women, Priests, and Physicians: Family Limitation in Quebec, 1940–1970’, *Journal of Interdisciplinary History*, 34 (2003), 293–314; Marloes Marrigje Schoonheim, *Mixing Ovaries and Rosaries Catholic Religion and Reproduction in the Netherlands, 1870–1970* (unpublished Ph.D. thesis, Radboud Universiteit Nijmegen, 2005); Máire Leane, ‘Embodied Sexualities: Exploring Accounts of Irish Women’s Sexual Knowledge and Sexual Experiences, 1920–1970’, in M. Leane and E. Kiely, eds, *Sexualities and Irish Society: A Reader* (Dublin: Orpen Press, 2014), 29–56; Betty Hilliard, ‘The Catholic Church and Married Women’s Sexuality: Habitus Change in late 20th Century Ireland’, *Irish Journal of Sociology*, 12 (2003), 28–49.

23 See: Caitriona Beaumont, ‘“The Housewife as Expert”: Re-Thinking the Experiential Expertise and Welfare Activism of Housewives’ Associations in England, 1960–1980’, in Caitriona Beaumont, Eve Colpus and Ruth Davidson, eds, *Everyday Welfare in Modern British History: Experience, Expertise and Activism* (Palgrave: 2025), 71–94; Grace Heaton ‘God is an Equal Opportunities Employer-Pity About the Church’: Humour and the Campaign for Women’s Ordination in the Church of England, 1978–1994’, *Women's History Review*, 34 (2025), 640–60.

24 On Irish feminist health activism, see, for example: Cara Delay and Beth Sundstrom, *Catching Fire: Women’s Health Activism in Ireland and the Global Movement for Reproductive Justice* (Oxford University Press, 2023) and Laura Kelly, ‘Irishwomen United, The Contraception Action Programme and the Feminist Campaign for Free, Legal, Contraception in Ireland, *c.*1975–1981’, *Irish Historical Studies*, 43 (2019), 269–97. On abortion rights activism in the 1980s, see: Mary McAuliffe, ‘Opening Pandora’s Box’: The Anti-Amendment Campaign, 1979–1983’, *Feminist Encounters: A Journal of Critical Studies in Culture and Politics*, 6 (2022), 1–13; Mary Muldowney, ‘Breaking the Silence: Pro-choice Activism in Ireland Since 1983’, in J. Redmond et al., eds, *Sexual Politics in Ireland* (Irish Academic Press, 2015), 127–53; Ann Rossiter, *Ireland's Hidden Diaspora: The Abortion Trail and the Making of a London-Irish Underground, 1980–2000* (IASC, 2009); Sandra McAvoy, ‘From Anti-Amendment Campaigns to Demanding Reproductive Justice: the changing landscape of Abortion rights activism in Ireland, 1983–2008’ in: J. Schweppe, ed, *The Unborn Child: Article 40.3.3. and Abortion in Ireland: Twenty-five years of Protection?* (Liffey Press, 2008), 15–45.

25 See: Tom Hesketh, *The Second Partitioning of Ireland: The Abortion Referendum of 1983* (Dublin: Brandsma Books, 1990); Cara Delay, ‘Wrong for Womankind and The Nation: Anti-Abortion Discourses in Ireland, 1967–1992’, *Journal of Modern European History*, 17 (2019), 312–25; Ruth Fletcher, ‘“Pro-life” Absolutes: Feminist Challenges, the Fundamentalist Narrative of Irish Abortion Law’, *Osgoode Hall Law Journal,* 36 (1998), 1–62 and ‘Post-colonial Fragments: Representations of Abortion in Irish Law and Politics’, *Journal of Law and Society,* 28 (2001), 568–89; Mary E. Daly, *The Battle to Control Female Fertility in Modern Ireland* (Cambridge University Press, 2023); Sylwia Kuźma-Markowska and Laura Kelly, ‘Anti-abortion Activism in Poland and the Republic of Ireland *c*.1970s–1990s’, *Journal of Religious History*, 46 (2022), 526–51. On the Irish Countrywomen’s Association, see: Diarmaid Ferriter, *Mothers, Maidens and Myths: A History of the Irish Countrywomen's Association* (Dublin: FÁS, 1995).

26 Cara Delay, *Irish Women and the Creation of Modern Catholicism* (Manchester University Press, 2019) and Lindsey Earner-Byrne, *Letters of the Catholic Poor: Poverty in Independent Ireland*, 1920–1940 (Cambridge University Press, 2017).

27 See, for example: Martina Avanza, ‘Using a Feminist Paradigm (Intersectionality) to Study Conservative Women: The Case of Pro-life Activists in Italy’, *Politics & Gender,* 16 (2020), 552–80 and Karissa Haugeberg, *Women Against Abortion: Inside the Largest Moral Reform Movement of the Twentieth Century* (University of Illinois Press, 2017).

28 Sylwia Kuzma-Markowska and Agata Ignaciuk, ‘Family Planning Advice in State-Socialist Poland, 1950s–80s: Local and Transnational Exchanges’, *Medical History*, 64 (2020), 240–66, on 266.

29 The project received ethical approval from the University of Strathclyde’s University Ethics Committee. Oral history interviews took place in 2020 and 2021. Due to the COVID-19 pandemic, all interviews were conducted on Zoom or by phone. Participants were audio recorded and sent a transcript of their recording following the interview for review. They had the option to be assigned a pseudonym, if desired, in the written record. Oral history interviews were analysed thematically with the assistance of NVivo software for coding.

30 The DOMAS minute books are currently held by Billings Method Ireland. The Mavis Keniry papers have recently been donated to the Royal College of Physicians of Ireland (RCPI) Heritage Centre, Dublin.

31 For a comprehensive overview of the history of the 1960s in Ireland, see: Mary E. Daly, *Sixties Ireland: Reshaping the Economy, State and Society, 1957–1973* (Cambridge University Press, 2016). For an account of Angela Macnamara, see: Paul Ryan, *Asking Angela Macnamara: An Intimate History of Irish Lives* (Dublin: Irish Academic Press, 2012).

32 Carole Holohan, *Reframing Irish Youth in the Sixties* (Liverpool University Press, 2018), 11.

33 Holohan, *Reframing Irish Youth in the Sixties*, 50.

34 Holohan, *Reframing Irish Youth in the Sixties*, 97.

35 Holohan, *Reframing Irish Youth in the Sixties*, 129.

36 Holohan, *Reframing Irish Youth in the Sixties*, 131.

37 See: Laura Kelly, *Contraception and Modern Ireland: A Social History, c.1922–92* (Cambridge University Press, 2023), 184–260.

38 Betty Hilliard, ‘The Catholic Church and Married Women’s Sexuality: Habitus Change in Late 20^th^ Century Ireland’, *Irish Journal of Sociology*, 12 (2003), 28–49, on 47.

39 Judith Harford and Jennifer Redmond, ‘I am Amazed at How Easily We Accepted It’: The Marriage Ban, Teaching and Ideologies of Womanhood in Post-Independence Ireland’, *Gender and Education*, 33 (2021), 186–201, on 199.

40 Irene Mosca and Robert E. Wright, ‘The Long-Term Consequences of the Irish Marriage Bar’, *The Economic and Social Review*, 51 (2020), 1–24, 3.

41 Louise Fuller, *Irish Catholicism Since 1950: The Undoing of a Culture* (Dublin: Gill & Macmillan, 2002), 240 and Chrystel Hug, *Chrystel Hug, The Politics of Sexual Morality in Ireland* (Basingstoke: Palgrave Macmillan, 1999), 110.

42 Mary E. Daly, *The Battle to Control Female Fertility in Modern Ireland*, 160.

43 Ailbhe Smyth, ‘The Women’s Movement in the Republic of Ireland, 1970–1990’, in Ailbhe Smyth, ed, *Irish Women’s Studies Reader* (Dublin, 1993), 245–69, 250.

44 See, for example: Patrick McDonagh, *Gay and Lesbian Activism in the Republic of Ireland, 1973–93*. (Bloomsbury Publishing, 2021) and Laura Kelly, ‘The Women's Right to Choose Group and Abortion Rights Activism in Early 1980s Dublin’, *Health and History*, 26 (2024), 51–72.

45 Diarmaid Ferriter, *Ambiguous Republic: Ireland in the 1970s* (London, 2012), 679.

46 Tom Hesketh, *The Second Partitioning of Ireland? The Abortion Referendum of 1983* (Dublin, 1990).

47 Hug, *Politics of Sexual Morality*, 121.

48 See Nell McCafferty, *A Woman to Blame: The Kerry Babies Case* (Dublin, 1985).

49 Laura Kelly, ‘The IFPA Youth Group, the Adolescent Confidential Telephone Service and Sexual Health Activism in Ireland, *c*. 1984–90’, *History Workshop Journal*, 98 (2024), 181–208, on 186.

50 DOMAS Teaching Associates meeting minutes, 9 May 1975.

51 ‘Why the Billings method is best’, *Sunday Independent*, March 26, 1978, 14.

52 DOMAS Executive meeting minutes, 5 October, 1979.

53 DOMAS Teachers’ meeting minutes, 14 November, 1975.

54 DOMAS Executive meeting minutes, 1 June 1979 and 2 November 1979.

55 DOMAS Executive meeting minutes, 2 November 1979.

56
*Natural Family Planning: Ovulation Method (Billings)*, (Cork: Ovulation Method Advisory Service, 1979).

57 The Ovulation Method: notes for instructors (1973), 1. [Mavis Keniry Papers].

58 DOMAS Teachers’ meeting minutes, 9 May 1975.

59 ‘Plea for nature birth plan’, *Irish Independent*, February 17, 1978, 7.

60 Oral history interview with Ita McDonnell, 10-12-2020.

61 ‘Women opt for Billings’, *Evening Herald*, September 17, 1980, 3.

62 ‘I.C.A. Family Planning Survey’, *The Irish Woman: The Official Journal of the Irish Countrywomen’s Association*, 1 (1978), 8–9 [Roisin Conroy/Attic Press archive: IF/AP/1492, UCC].

63 Minutes of 1986 AGM, 27 April 1986. [Mavis Keniry Papers].

64 DOMAS Teachers’ meeting minutes, 8 October 1976.

65 Oral history interview with Vera O’Donoghue, 07-12-2020.

66 The Ovulation Method: notes for instructors (1973), 1. [Mavis Keniry Papers].

67 Vera O’Donoghue.

68 ‘Accreditation of Ovulation Method Teachers’, undated but likely late 1970s or early 1980s. [Mavis Keniry].

69 Oral history interview with Anne Shinners, 02-12-2020.

70 Oral history with Noreen Agar (pseudonym), 01-12-2020.

71 DOMAS Teachers’ meeting minutes, 13 June 1975.

72 DOMAS Teachers’ meeting minutes, 13 June 1975.

73 DOMAS Teachers’ meeting minutes, 11 July 1975.

74 Constitution for National Association of the Ovulation Method of Ireland (NAOMI), [Mavis Keniry Papers].

75 DOMAS Executive meeting minutes, April 6, 1979.

76 DOMAS Teachers’ meeting minutes, 10 March 1978.

77 Oral history interview with Mary (pseudonym), 04-08-2021.

78 Vera O’Donoghue.

79 Vera O’Donoghue.

80 David Geiringer, ‘Biofaiths: Catholic Young Women and the Generation of Sexual Knowledge in Post-War England’, *Religion and Gender*, 15 (2025), 290–310, on 294.

81 Kelly, *Contraception and Modern Ireland*, 123–27.

82 Oral history interview with Niamh (pseudonym), 27-01-21.

83 Oral history interview with Therese Shorten, 21-01-2021.

84 Oral history interview with Mena (pseudonym), 30-11-2020.

85 Noreen Agar.

86 Oral history interview with Maura (pseudonym), 01-11-2021.

87 Deirdre Foley, ‘“Their Proper Place”: Women, Work and the Marriage Bar in Independent Ireland, c.1924– 973’, *Social History*, 47 (2022), 60–84, on 83–84.

88 Mary.

89 Mary.

90 Vera O’Donoghue.

91 Therese Shorten.

92 Vera O’Donoghue.

93 Oral history interview with Veronica Kendrick, 22-01-2021.

94 Anne Shinners.

95 Anne Shinners.

96 Mena.

97 Noreen Agar.

98 Niamh.

99 Maura.

100 Mena.

101 Maura.

102 Heather Parsons, ‘Why Not the Natural Way?’, *Woman’s Way*, April 23, 1982, 10–14.

103 Mena.

104 See: Alana Harris, *Faith in the Family: a lived religious history of English Catholicism, 1945–82* (Manchester University Press, 2013), 130–201.

105 DOMAS Teachers’ meeting minutes, 11 July, 1975 and 10 October, 1975.

106 DOMAS Teachers’ meeting minutes, 9 September, 1977.

107 Maura.

108 Ita McDonnell.

109 DOMAS Executive meeting minutes, April 6, 1979.

110 DOMAS Teachers’ meeting minutes, 14 January 1977.

111 DOMAS Teachers’ meeting minutes, 11 February 1977.

112 ‘Natural family planning’, *Connacht Tribune*, February 3, 1978, 31.

113 DOMAS Executive meeting minutes, 6 February 1981.

114 DOMAS Executive meeting minutes, 17 December 1981.

115 ‘Billings trip: itinerary’, June 1985' [Mavis Keniry Papers].

116 ‘D.O.M.A.S.: Lists of persons to be invited to public lecture, 6 June 1985’. [Mavis Keniry Papers].

117 Minutes of 1986 N.A.O.M.I. AGM, 27 April 1986. [Mavis Keniry Papers].

118 Letter from John Billings to Mavis Keniry, 23 May 1987. [Mavis Keniry Papers].

119 ‘Obituary: St. Maureen O’Sullivan’, Medical Missionaries of Mary website: https://mmmworldwide.org/obituaries/o-sullivan-sr-maureen/

120 Eileen.

121 Oral history interview with Maud (pseudonym), 02-04-2021.

122 Oral history interview with Jean (pseudonym), 26-03-2021.

123 ‘Lecture’, *Connacht Tribune*, July 15, 1977, 19.

124 Vera O’Donoghue.

125 DOMAS Teachers’ meeting minutes, 14 November, 1975 and 20 August, 1976.

126 DOMAS Teachers’ meeting minutes, 9 September 1977.

127 DOMAS Executive meeting minutes, 4 June 1982.

128 Oral history interview with Eileen (pseudonym), 13-04-2021.

129 Máire Leane, ‘Embodied Sexualities: Exploring Accounts of Irish Women's Sexual Knowledge and Sexual Experiences, 1920–1970’, in Máire Leane and Elizabeth Kiely, eds, *Sexualities and Irish Society: A Reader* (Dublin: Orpen, 2014), 29–56, on 53.

130 Harris, ‘A Magna Carta for Marriage’, 423.

131 Ita McDonnell.

132 Mary.

133 Ita McDonnell.

134 Noreen Agar.

135 DOMAS Teachers’ meeting minutes, 12 September 1975.

136 Anne Shinners.

137 Eileen.

138 NAOMI, *Excerpts from Billings Atlas of the Ovulation Method: the Mucus Patterns of Fertility and Infertility, Fifth edition 1989* by Evelyn Billings, John Billings and Maurice Catarinich (for private circulation to N.A.O.M.I. teachers only), 2. [Mavis Keniry papers].

139 ‘Letters to the editor’, *Irish Times*, July 15, 1981, 9.

140 ‘Natural family planning gets state boost’, *Irish Independent*, March 18, 1981, 14.

141 Oral history interview with Sarah (pseudonym), 12-03-2021.

142 Jean.

143 In Poland, the Society of Conscious Motherhood began to promote fertility awareness techniques such as the Billings Method due to the ‘increasing standing of the Church, and its alignment with the party-state vision of marriage, family and fertility’ but also due to the economic crisis which meant that ‘teaching fertility awareness, which did not require any product or device, perhaps proved the most practical family planning system in this complicated context.’ Kuzma-Markowska and Ignaciuk, ‘Family Planning Advice in State-Socialist Poland’, 266.

144 ‘The doctor’s dilemma’, *Irish Independent*, March 9, 1979, 8.

145 Niamh.

146 ‘Letters to the editor’, *Irish Times*, February 13, 1976, 6.

147 Lara Marks, *Sexual Chemistry: a history of the contraceptive pill*, (Yale University Press, 2001), 138–57.

148 Marks, *Sexual Chemistry*, 138.

149 Elizabeth Siegel Watkins, *On the Pill*, 104 and 119.

150 Shirley Johnson, ‘Why they came off the pill’, *Woman’s Way*, March 23, 1979, 12–14.

151 ‘Letters to the editor’, *Evening Herald*, May 2, 1979, 23.

152 Statement by Irishwomen United. [Roisin Conroy/Attic Press archive, University College Cork: BL/F/AP/1177/23].

153 Oral history interview with Sonya Keniry, 03-12-2020.

154 ‘Billings method – a blessing from heaven?’, *Evening Herald*, December 14, 1973, 37.

155 ‘Family Planning’, *Connacht Tribune*, April 21, 1978, 9.

156 ‘Letters to the editor’, *Connacht Tribune*, November 18, 1977, 9. The author is stated to be J. Manifold but I am confident this is an error.

157 NAOMI, *Excerpts from Billings Atlas of the Ovulation Method: the Mucus Patterns of Fertility and Infertility, Fifth edition 1989* by Evelyn Billings, John Billings and Maurice Catarinich, (for private circulation to N.A.O.M.I. teachers only, 18. [Mavis Keniry papers].

158 Agata Ignaciuk, ‘Regendering Childbirth: Catholicism, Medical Activism, and Birth Preparation in Post-War Poland’, *Journal of the History of Medicine and Allied Sciences*, 78 (2023), 249–69, on 268.

159 Avanza, ‘Using a Feminist Paradigm (Intersectionality) to Study Conservative Women’, 554.

160 Alana Harris, ‘A Magna Carta for Marriage’, 411.

161 Mary Leland, ‘Billings Comes to Cork’, *Irish Times*, January 22, 1973, 12.

162 Alana Harris, ‘Love Divine and Love Sublime: The Catholic Marriage Advisory Council, the Marriage Guidance Movement and the State’, in Alana Harris et al., eds, *Love and Romance in Britain, 1918–1970*, (Palgrave Macmillan, 2015), 188–224, on 216.

163 Natalia Jarska and Agata Ignaciuk, ‘Marriage, Gender and Demographic Change: Managing Fertility in State-Socialist Poland’, *Slavic Review*, 81 (2002), 142–62, on 161.

164 Oral history interview with Mary, 04-08-2021.

165 Alana Harris, ‘A Magna Carta for Marriage: Love, Catholic Masculinities and the Humanae Vitae Contraception Crisis in Britain’, *Cultural and Social History*, 17 (2020), 407–429, on 412.

166 Jarska and Kuzma-Markowska, ‘Explaining the Calendar’, 682.

167 Agata Ignaciuk, ‘Regendering Childbirth: Catholicism, Medical Activism, and Birth Preparation in Post-War Poland’, *Journal of the History of Medicine and Allied Sciences*, 78 (2023), 249–69, On 260–61.

168 ‘Help page’, *Woman’s Way*, December 6, 1974, 68.

169 Rev. Dermot Hurley, ‘Evaluating Reports of Ovulation Method Effectiveness’, (1976), 13. [Mavis Keniry Papers.]

170 DOMAS Executive meeting minutes, 2 November 1979.

171 DOMAS Executive meeting minutes, 7 March 1980.

172 ‘Late, Late Billings row’, *Irish Examiner*, November 20, 1981, 24.

173 Ita McDonnell.

174 DOMAS Teachers’ meeting minutes, 12 September 1975.

175 DOMAS Teachers’ meeting minutes, 14 November 1975.

176 DOMAS Teachers’ meeting minutes, 9 April 1976.

177 DOMAS Executive meeting minutes, 6 March 1981.

178 DOMAS Teachers’ meeting minutes, 11 February 1977.

179 DOMAS Teachers’ meeting minutes, 6 April 1977.

180 ‘Doctor’s view on family planning’, *Irish Times*, May 15, 1979, 4.

181 Alana Harris, ‘Love Divine and Love Sublime: The Catholic Marriage Advisory Council, the Marriage Guidance Movement and the State’, in Alana Harris et al., eds, *Love and Romance in Britain, 1918–1970* (Palgrave Macmillan, 2015), 188–224, on 206.

182 DOMAS Teachers’ meeting minutes, 20 August 1976.

183 DOMAS Teachers’ meeting minutes, 17 September 1976.

184 DOMAS Teachers’ meeting minutes, 10 February 1978.

185 DOMAS Teachers’ meeting minutes, 4 May, 1979.

186 DOMAS Teachers’ meeting minutes, 13 May 1977.

187 DOMAS Teachers’ meeting minutes, 2 November 1979.

188 DOMAS Executive meeting minutes, 7 March 1980.

189 Mena.

190 DOMAS Executive meeting minutes, 6 February, 1981.

191 Letter to parish priests, dated 13 January 1986. [Mavis Keniry Papers].

192 For a recent edited collection on Catholic lay societies in Britain which explores the tensions and rivalries between these see: Maria Power and Jonathan Bush, eds, *Catholic Lay Societies in Twentieth Century Britain* (Boydell and Brewer, 2025).

193 DOMAS Executive meeting minutes, 6 April, 1984.

194 Ita McDonnell.

195 Delay, *Irish Women and the Creation of Modern Catholicism*, 239.

196 DOMAS Executive meeting minutes, 14 December 1979.

197 DOMAS Teachers’ meeting minutes, 7 March 1980.

198 DOMAS Executive meeting minutes, 2 May 1980.

199 Letter from Monsignor Alex Stenson, ‘Dublin Diocesan Council for the Family’, dated 7 December 1988, 4726.

200 DOMAS Teachers’ meeting minutes, 11 April 1975.

201 DOMAS Teachers’ meeting minutes, 14 November 1975.

202 Minutes of DOMAS Teaching Associates meeting, 12 November 1976.

203 Letter from Mavis Keniry to Archbishop Desmond Connell, dated 5 January 1989. [Mavis Keniry papers]. 4732

204 Veronica Kendrick.

205 DOMAS Teachers’ meeting minutes, 9 April 1976.

206 DOMAS Teachers’ meeting minutes, 17 September 1976.

207 DOMAS Executive meeting minutes, 2 May 1980.

208 DOMAS Executive meeting minutes, 6 June 1980.

209 DOMAS Executive meeting minutes, 5 March 1982.

210 Hug, *The Politics of Sexual Morality in Ireland*, 146.

211 Chrystel Hug, *The Politics of Sexual Morality in Ireland* (Basingstoke: Palgrave Macmillan, 1998), 154.

212 Hug, *The Politics of Sexual Morality in Ireland,* 155.

213 DOMAS Executive meeting minutes, 6 February 1981.

214 For a detailed study of the PLAC, see: Tom Hesketh, *The Second Partitioning of Ireland: The Abortion Referendum of 1983* (Brandsma Books, 1990).

215 DOMAS Executive meeting minutes, 5 June 1981.

216 DOMAS Executive meeting minutes, 5 June 1981.

217 DOMAS Executive meeting minutes, 30 July 1981.

218 DOMAS Executive meeting minutes, 5 March 1982.

219 DOMAS Executive meeting minutes, 3 September 1982.

220 DOMAS Executive meeting minutes, 4 March 1983.

221 DOMAS Teachers’ meeting minutes, 10 February 1978.

222 DOMAS Executive meeting minutes, 9 February 1979.

223 DOMAS Executive meeting minutes, 6 February 1981.

224 DOMAS Executive meeting minutes, 30 July 1981.

225 DOMAS Executive meeting minutes, 6 April 1979, 4 May 1979, 5 March 1982, 6 January 1984.

226 Minutes of N.A.O.M.I. 1989 AGM, dated 30 April 1989. [Mavis Keniry Papers].

227 Veronica Kendrick.

228 Laury Oaks, ‘Feminist Fertility Awareness? Sexual and Reproductive Knowledge in a Post-Roe World’, *Signs,* 49 (2024), 859–85, on 875.

229 Maura.

